# Malignant phyllodes tumor of the breast with short-term multiple recurrences and systemic distant metastasis: a case report and literature review

**DOI:** 10.3389/fonc.2025.1715674

**Published:** 2025-12-11

**Authors:** Min Zhang, Lisha Zhou, Xudong Gao, Mengfan Zhao, Rupei Ye, Bin Liang, Bin Wu

**Affiliations:** 1 Department of Breast Surgery, The Affiliated Hospital of Southwest Medical University, Luzhou, China; 2 Department of Pathology, The Affiliated Hospital of Southwest Medical University, Luzhou, China

**Keywords:** phyllodes tumor, breast sarcoma, recurrence, metastasis, diagnosis, therapy

## Abstract

Malignant phyllodes tumor (MPT) of the breast is a rare fibroepithelial neoplasm that is often misdiagnosed as a benign tumor or fibromatosis. It is characterized by rapid growth and a high tendency for recurrence, though distant metastasis is relatively uncommon. This case report presents a 61-year-old female patient with a breast mass that was initially suspected to be a fibroadenoma based on core needle biopsy. Consequently, she underwent lumpectomy, and postoperative pathology indicated a tendency towards desmoid-type fibromatosis. However, the tumor recurred rapidly 29 days later, prompting a subsequent total mastectomy. The final diagnosis confirmed malignant phyllodes tumor. Forty days later, during radiotherapy, the tumor recurred again with concurrent systemic distant metastasis, and the patient was subsequently initiated on systemic therapy. By analyzing the diagnostic and treatment process as well as pathological features, this report highlights the importance of early definitive diagnosis, surgical margins, adjuvant therapy, and systemic treatment in improving the prognosis of breast MPT.

## 1 Introduction

Phyllodes tumor (PT) of the breast is an uncommon fibroepithelial neoplasm, accounting for 0.3% to 1% of all primary breast tumors. According to the World Health Organization classification(WHO), PT are categorized into benign, borderline, and malignant types based on histological features including nuclear atypia, stromal cellularity, mitotic activity, tumor margin appearance, and stromal overgrowth. All types of PT are capable of recurrence, with local recurrence rates of approximately 8%, 21%, and 30% for benign, borderline, and MPT, respectively (1). A systematic review of malignant PT by Samii E et al. reported that local-regional recurrence was observed at a median time of 8.9 months, while metastatic recurrence occurred at a median of 9.0 months (2). In the present case, however, the MPT exhibited highly aggressive behavior, recurring locally within just 29 days. Despite undergoing total mastectomy and radiotherapy after the first recurrence, the tumor recurred again approximately 40 days, accompanied by distant metastasis.

## 2 Case description

**Figure 1:**
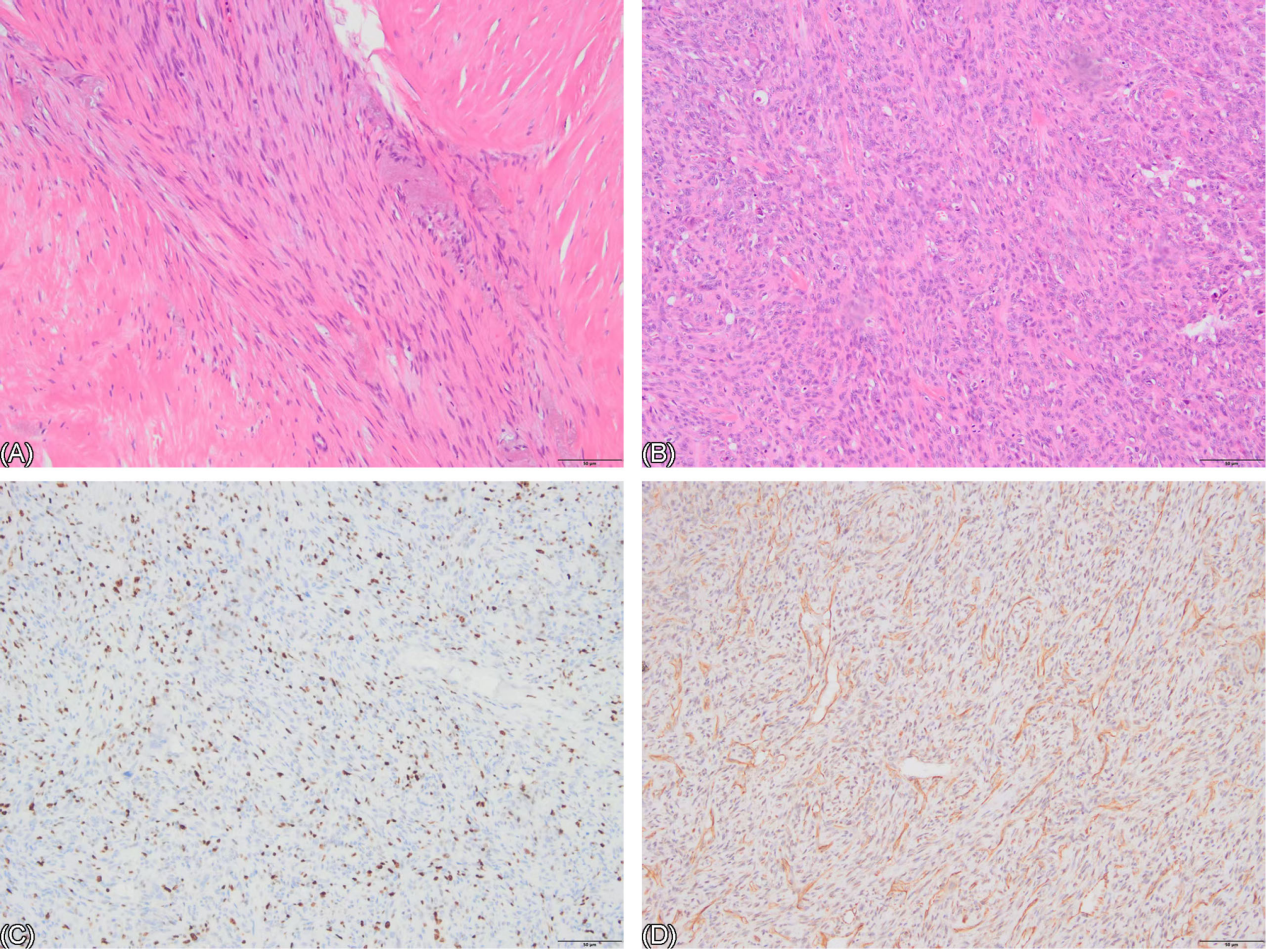
**(A, B)** HE staining of the biopsy specimen. **(A)** shows numerous spindle cells arranged in fascicles, exhibiting bland morphology and rare mitotic figures. The cells are separated by abundant collagen fibers, with no epithelial structures observed (x100 magnification). **(B)** reveals cells predominantly composed of short spindle and irregular shapes, arranged in a disordered pattern with high cellular density. Mitotic figures are rare, and no epithelial structures are identified (x100 magnification). **(C, D)** Immunohistochemistry. **(C)** shows a brown positive signal localized to the nucleus, representing the Ki-67 marker (an indicator of cellular proliferative activity), with approximately 20% positivity. **(D)** displays a brownish-yellow positive signal localized to the stroma, indicating positive Vimentin staining (VIM+).

The patient is a 61-year-old female, presented with a chief complaint of a left breast mass discovered over 10 years ago, which had shown enlargement for more than 4 months. She was admitted to the Affiliated Hospital of Southwest Medical University on October 18, 2024. A timeline summarizing key diagnostic and treatment points is provided in **Table 1**. Initial physical examination revealed a hard, fixed, irregular mass approximately 5.0 × 5.0 cm in the upper outer quadrant of the left breast. Breast ultrasound identified an irregular, lobulated, hypoechoic mass measuring approximately 5.0 × 4.5 × 3.8 cm at the 9–2 o’clock position, assessed as BI-RADS 4A. Mammography demonstrated a high-density shadow measuring about 3.9 × 3.3 cm, assessed as BI-RADS 5. Routine laboratory tests showed no significant abnormalities. there is no family history of malignancy. Core needle biopsy pathology suggested a fibroadenoma with hyaline degeneration. Given the benign nature indicated by the biopsy, preoperative assessments were performed to rule out surgical contraindications. Subsequently, on November 7, 2024, the patient underwent a lumpectomy of the left breast mass. Postoperative pathological examination indicated a mesenchymal-origin tumor and areas of invasive growth, suggestive of desmoid-type fibromatosis. Immunohistochemistry results were as follows: Vim(+), ER (–), SAM (–), Desmin (–), β-catenin (–), CD34 (–), P53 (wild type), Ki-67 (20%), STAT6 (–), CK (–), CD117 (–), PR (–)(**Figure 1**).Desmoid-type fibromatosis is a benign lesion primarily treated by surgical excision. Due to its tendency for local recurrence, the patient was placed under close follow-up observation. However,29 days postoperatively, the patient noticed induration around the surgical incision. Follow-up breast ultrasound revealed multiple irregular hypoechoic masses. Initially considered hematomas, and continued observation was recommended. As the patient felt progressive enlargement of the mass, a repeat ultrasound was performed. It showed multiple irregular and lobulated hypoechoic masses in the left breast, assessed as BI-RADS 4C. Mammogram revealed a high-density shadow, measuring about 8.1 × 5.4 cm, with poorly defined borders and an irregular shape, classified as BI-RADS 5. Breast MRI demonstrated multiple irregular soft-tissue signal masses and nodules. Enhanced scanning showed heterogeneous enhancement with non-enhancing necrotic areas. The largest cross-section measured approximately 8.2 × 4.4 cm. The findings were highly suggestive of recurrent aggressive fibromatosis. Further examinations, including chest CT and abdominal ultrasound, showed no evidence of distant metastasis.

**Table 1:** Clinical timeline of diagnosis, treatment, and disease progression.

Time	Time from previous event	Key event/finding	Clinical decision/outcome
10+ years ago	–	Patient discovered a lump in the left breast, approximately the size of a “little fingertip”.	Observation.
2024-06	–	The lump began to grow rapidly.	Observation.
2024-10-18	About 110+ days	Hospital visit. Palpable mass (5 cm) in left breast. Imaging (Ultrasound: BI-RADS 4A; Mammogram: BI-RADS 5). Core biopsy: Fibroadenoma.	Planned for excisional biopsy.
2024-11-07	19 days	First Surgery: Left breast lumpectomy.	Post-operative diagnosis: Desmoid-type fibromatosis. Plan: follow-up observation.
2024-12-06	29 days	Palpable induration at incision site. Ultrasound: Possible hematoma.	Continued observation.
2025-01-22	47 days	The induration enlarging. Imaging (Ultrasound: BI-RADS 4C. Mammogram: BI-RADS 5. MRI: Multiple irregular masses, suspected recurrence).	Suspected recurrence. Planned for second hospitalization and surgery.
2025-02-08	17 days	Second Surgery: Left mastectomy + latissimus dorsi flap.	Final Diagnosis: Malignant Phyllodes Tumor. Plan: Adjuvant radiotherapy.
2025-03-20	40 days	Local Recurrence and Metastasis: Palpable nodules on chest wall during radiotherapy. PET/CT confirms widespread systemic metastases.	Radiotherapy paused. Systemic chemotherapy initiated (Doxorubicin + Docetaxel).
2025-04-23	34 days	The treatment resulted in severe myelosuppression and an insufficient therapeutic outcome	Regimen switched to Gemcitabine + Cisplatin + Anlotinib.
2025-07	About 68+ days	The patient died	

**Figure 2:**
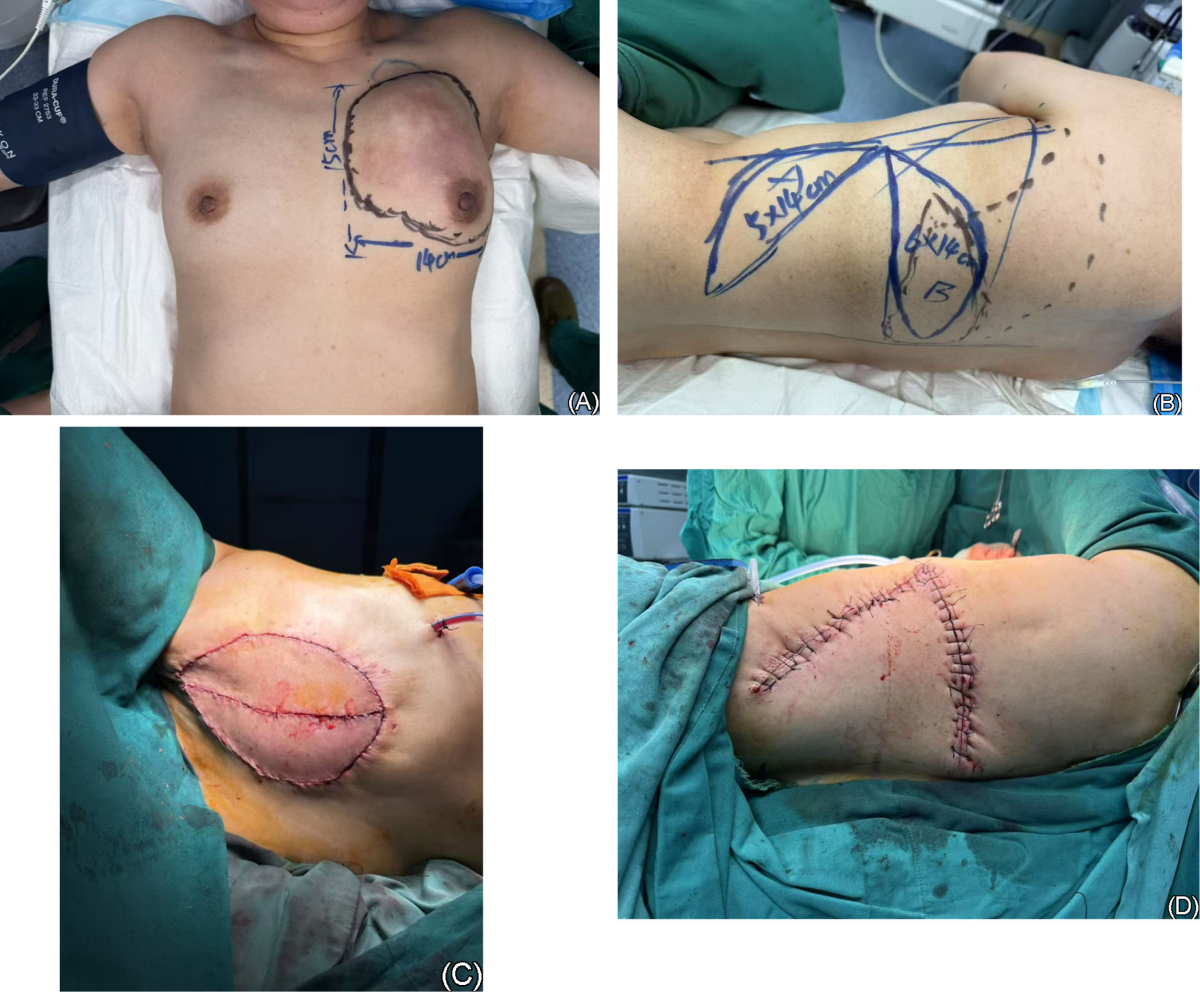
**(A)** Marking the size and location of the mass, and designing the incision. **(B)** Marking the size and location of the latissimus dorsi myocutaneous flap to be utilized, and designing the incision. **(C)** Postoperative photograph of the anterior chest wall, showing the formation of a “Kissing” flap. **(D)** Postoperative photograph of the back.

**Figure 3:**
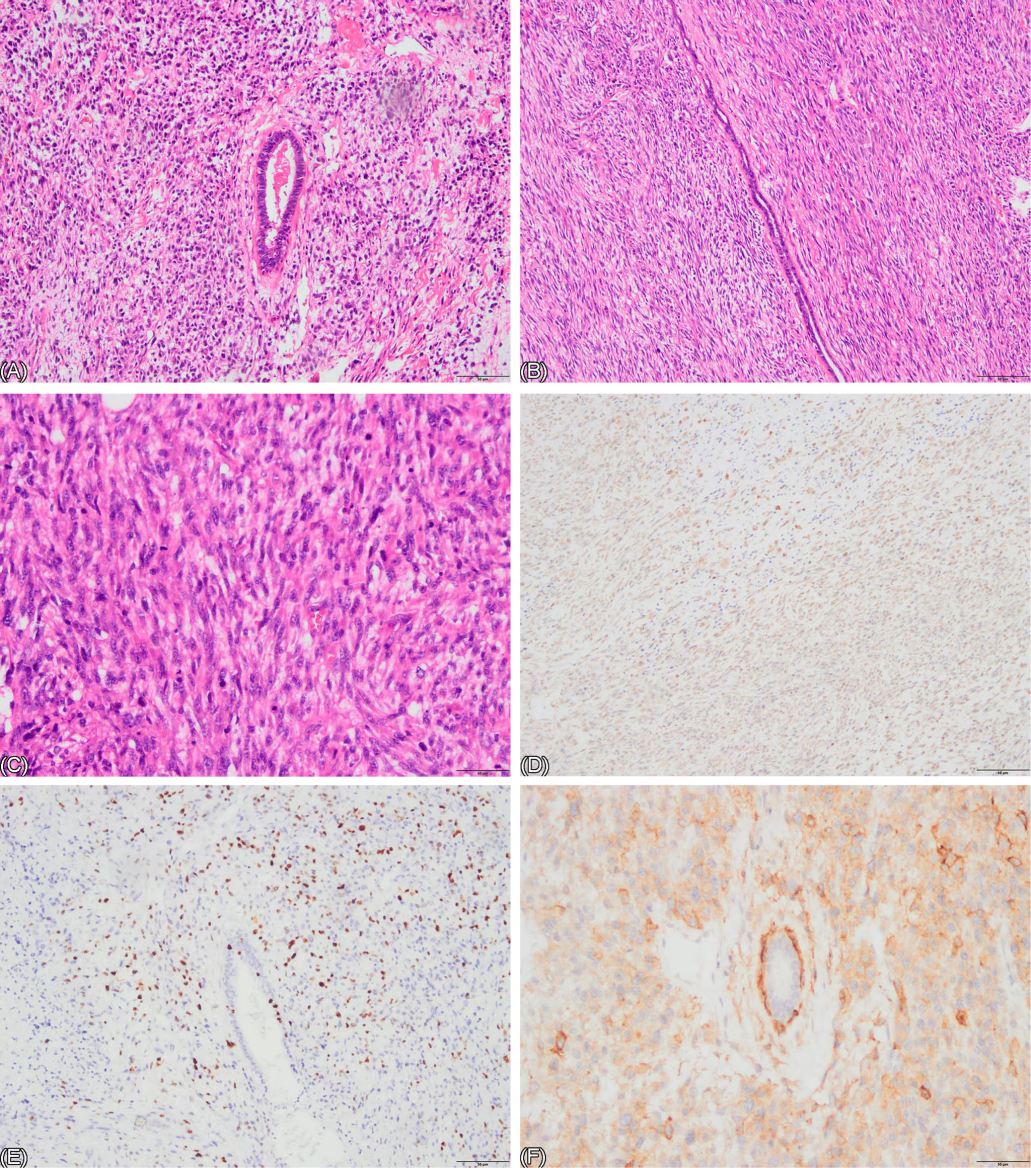
**(A–C)** HE staining of the biopsy specimen. **(A)** demonstrates both stromal components (spindle cells) and epithelial components (glandular ducts). Stromal overgrowth is evident, with a marked reduction in the epithelial component. The stromal cells are densely arranged in a diffuse pattern, exhibiting significant atypia. (×100). **(B)** shows stromal overgrowth with cells arranged in fascicles or a diffuse pattern, displaying marked cellular atypia. (×100). **(C)** illustrates stromal overgrowth with high cellular density, significant atypia, and numerous mitotic figures. (×200). **(D–F)** Immunohistochemistry: **(D)** shows brownish-yellow positive staining in tumor cells, indicating mesenchymal origin, consistent with VIM positivity (VIM+). **(E)** displays brown positive nuclear staining in tumor cells, with a positivity rate of approximately 40%, representing Ki-67 expression. **(F)** exhibits brown positive staining in the stromal region of the tumor, indicating CD-10 positivity (CD-10+).

Desmoid-type fibromatosis is characterized by infiltrative growth and a tendency for local recurrence if incompletely excised, but it does not metastasize. Based on the patient’s history and current auxiliary examinations, the condition was considered most consistent with recurrent desmoid-type fibromatosis. However, due to the large size of the tumor and the difficulty in achieving complete excision, the case was discussed within the department. Subsequently, on February 8, 2025, the patient underwent left mastectomy with latissimus dorsi pedicled flap transplantation (**Figure 2**). During the procedure, the tumor was found to be densely adherent to the skin and portions of the pectoralis major muscle. As a result, the overlying skin and involved parts of the pectoralis major muscle along with its fascia were resected en bloc. Due to the extensive defect, primary skin closure was not feasible. A pedicled latissimus dorsi myocutaneous flap was therefore transposed to the defect site, forming a “kissing” flap. Postoperative pathological findings included: Tumor size: approximately 10.3 × 8.6 × 4.9 cm; Histologic type: consistent with MPT, supported by immunohistochemical results; Surgical margins: no tumor involvement was identified at the peripheral or basal margins; Lymphovascular invasion: not identified; Perineural invasion: present; Nipple and skin involvement: not identified. Immunohistochemistry results were as follows:

Vim(+), CD10(+), CD117 (focal+), EMA (–), CD34 (–), STAT6 (–), SMA (–), Desmin (–), S100 (–), CD31 (–), ERG (–), ER (–), CAM5.2 (–), β-catenin (–), P53 (+, 40%), Ki-67 (40%) (**Figure 3**). The patient’s diagnosis has now been revised to MPT. The most effective treatment for MPT is surgical resection with a recommended margin of at least 1 cm. In this case, the achieved surgical margin significantly exceeded 1 cm. Since PT rarely metastasize to axillary lymph nodes, and no enlarged or suspicious lymph nodes were detected in preoperative assessments, sentinel lymph node biopsy was deemed unnecessary. Therefore, no further extended resection or lymph node procedure was indicated. Although PT are generally insensitive to both chemotherapy and radiotherapy, considering the patient’s multiple recurrences, localized adjuvant radiotherapy was planned post wound healing, while systemic chemotherapy was not initially recommended. However, during radiotherapy (on March 20, 2025), induration reappeared around the incision. Two masses, approximately 2×2 cm and 1×1 cm in size, were palpated near the sternum. A subsequent PET/CT scan revealed multiple metastatic lesions throughout the body, including the anterior chest wall, left pectoral muscle interstice, bilateral axillary lymph nodes, multiple pulmonary nodules, the axis vertebra, and the humerus (among other sites). A fine-needle aspiration biopsy of a right axillary lymph node confirmed a spindle cell tumor. Following a multidisciplinary team discussion, the condition was assessed as another recurrence of malignant phyllodes tumor with widespread systemic metastasis. Salvage chemotherapy with doxorubicin + docetaxel was initiated (Doxorubicin 100mg + Docetaxel 100mg). After the first cycle, the patient’s sternal masses enlarged (the larger one to approximately 4x4 cm, the smaller to about 1.5x1.5 cm), indicating disease progression. Furthermore, the patient developed grade IV myelosuppression (white blood cell 1.47x10^9/L, neutrophils 0.4x10^9/L). Consequently, the treatment regimen was changed to a combination of Gemcitabine + Cisplatin chemotherapy along with the targeted therapy Anlotinib (Cisplatin 100mg + Gemcitabine total 1200mg + Anlotinib 8mg orally). After one cycle of this new regimen, the patient declined further treatment due to personal reasons, including significant side effects such as vomiting, lack of apparent short-term efficacy, and the cumulative burden of multiple chemotherapy cycles. The patient subsequently passed away in July 2025.

## 3 Discussion

This case report illustrates a MPT of the breast with an exceptionally rare biological behavior, marked by extremely short recurrence intervals and rapid systemic dissemination. We will discuss this case in detail, exploring strategies for early definitive diagnosis, standardized treatment, and improved prognosis for MPT.

### 3.1 Imaging characteristics

Both at initial presentation and the subsequent visit for recurrence, imaging modalities including ultrasound, mammography, and MRI consistently revealed lobulated, irregular, ill-defined masses, categorized as BI-RADS 4A or higher, suggesting a high probability of malignancy radiologically. However, these findings were non-specific and easily confused with other tumors, thus preventing a definitive preoperative diagnosis. Through this case and related literature, we learn that imaging features such as large mass size (especially >3cm), lobulated shape, irregular borders, internal heterogeneity, necrosis, cystic spaces, and increased vascularity should raise suspicion for phyllodes tumor (3). Studies also show potential for deep learning-based radiomic models in differentiating PT from fibroadenoma (4). Nonetheless, while preoperative imaging serves as the foundation for the initial assessment of PT, the final definitive diagnosis must rely on pathological examination.

### 3.2 Pathological characteristics

The patient’s initial core needle biopsy suggested fibroadenoma with hyalinization. Core needle biopsy is a standard preoperative diagnostic tool. however, its accuracy in diagnosing PT is relatively low, reportedly only 13.3% (5). The pathology from the first lumpectomy favored desmoid fibromatosis. Some pathological slides revealed abundant spindle cells arranged in fascicles, exhibiting bland morphology and rare mitotic figures. The cells were separated by abundant collagen fibers, with no epithelial structures observed. Some slides showed cells predominantly composed of short spindle and irregular shapes, arranged in a disordered pattern with high cellular density, though mitotic figures remained rare. Immunohistochemical staining was positive for Vimentin, indicating mesenchymal origin. Ki-67, a marker of cellular proliferative activity, was approximately 20%, indicating relatively high proliferative activity. Consequently, the diagnosis was considered to be desmoid-type fibromatosis. Prior to the mastectomy for the first recurrence, the pathological slides from the initial lumpectomy were reviewed, and a diagnosis of sarcoma could not be ruled out, warranting molecular testing for clarification. Following the mastectomy, the diagnosis was confirmed as MPT. Pathological examination of the sections revealed both epithelial components (glandular ducts) and stromal components (spindle cells). Stromal overgrowth was evident, with a marked reduction in the epithelial component. The stromal cells were densely arranged in fascicles or a diffuse pattern, exhibiting significant cellular atypia and frequent pathological mitotic figures. One or two slides showed compressed, elongated ducts (cleft-like ducts). Immunohistochemistry showed widespread positivity for stromal markers VIM(+) and CD10(+), high proliferation index (Ki-67 40%), and abnormal high expression of P53 (+, 40%), all supporting the diagnosis of MPT. Thus, the diagnosis was finally confirmed. PT are fibroepithelial lesions comprising both stromal and epithelial components, each capable of exhibiting diverse histopathological alterations. PT resemble intracanalicular fibroadenomas, with bilayered epithelial components lining leaf-like clefts surrounded by hypercellular stroma. WHO classifies PT into benign, borderline, and malignant categories. The diagnosis of malignant phyllodes tumor requires the presence of the following features: infiltrative borders, diffusely hypercellular stroma, significant stromal nuclear atypia, increased mitotic activity (≥10 per 10 high-power fields, HPF), and stromal overgrowth. Stromal overgrowth is defined as the absence of epithelial elements in at least one microscopic field at 40x magnification (using a 4x objective and 10x eyepiece). A diagnosis of borderline PT is made when only some of these criteria are met. Benign PT feature cellular stroma with uniform spindle cells, rare mitotic figures (<5 per 10 HPF), and typically well-circumscribed borders (6).

### 3.3 Surgical scope and importance of margins

Surgical excision is the primary treatment for PT. The fundamental principle of local management for all PT types is complete excision with negative margins to achieve effective local control, as the status of the surgical margin is a key determinant of local recurrence risk (6). Positive margins are strongly associated with a significantly elevated risk of local recurrence, regardless of tumor grade. Consequently, the National Comprehensive Cancer Network (NCCN) guidelines recommend a margin of at least 1 cm or more as the optimal approach for conservative surgery (7). However, some studies suggest negative margin may be sufficient for benign PT, whereas for borderline and especially malignant PT, pursuing wide margins ≥1 cm is a widespread consensus to minimize recurrence risk (8). In this case, the patient experienced rapid recurrence shortly after the initial lumpectomy. Although no explicit positive margin was documented, the aggressive biological behavior suggests the initial surgery likely failed to achieve a true “wide margin”. Following the first recurrence, the patient underwent a total mastectomy. Intraoperatively, when the tumor was found adjacent to the chest wall or skin, the involved skin and partial muscle were also resected, resulting in surgical margins significantly exceeding 1 cm. Despite this, the tumor recurred again within a short period, accompanied by systemic metastasis. This rare and striking clinical course underscores a critical point: for certain MPT with highly aggressive biological behavior, even macroscopically adequate wide margins may be insufficient to control disease progression. Therefore, for tumors harboring high-risk features—such as moderate to severe stromal atypia, hypercellularity, stromal overgrowth, mitotic count >5/10 HPF, tumor necrosis, infiltrative tumor borders, and positive margins—the risk of recurrence and metastasis is substantially increased (9). While pursuing wide margins, we must also fully recognize the risk of systemic recurrence and actively consider comprehensive treatment, including radiotherapy, chemotherapy, and targeted therapy, to synergistically improve outcomes.

### 3.4 Radiotherapy efficacy and indications

Evidence for adjuvant radiotherapy in MPT remains insufficient, however, available data support its positive role in local control (10). Larger and more robust studies are needed to definitively confirm the benefits of radiotherapy. It is generally not considered an intervention that improves overall survival. The primary indications for radiotherapy are targeted at high-risk patients, such as those undergoing breast-conserving surgery, with tumor diameter >5 cm, stromal overgrowth, mitotic count >10/10 HPF, or close/positive margins where further wide excision is not feasible (7) (11). In this case, post-mastectomy chest wall radiotherapy was administered precisely based on the presence of high-risk features: the massive tumor size (>10 cm), stromal overgrowth, and a history of rapid recurrence within a short period. This adjuvant treatment was intended to consolidate local control. Unfortunately, the tumor progressed during the radiotherapy course, further confirming its highly aggressive nature.

### 3.5 Chemotherapy regimens

Approximately 9-27% of patients with MPT develop distant metastases. Hematogenous spread most commonly occurs to sites such as the lungs, bones, and brain, and rarely to organs like the liver and heart. Regional lymph node enlargement is common but rarely due to metastatic PT (12). Chemotherapy is currently employed for recurrent and metastatic disease, however, no standardized chemotherapy regimen exists. Recurrent or metastatic PT is typically managed according to NCCN guidelines for metastatic soft tissue sarcoma (5). Patients often receive doxorubicin-based chemotherapy as first-line treatment, either as monotherapy or in combination. Combination chemotherapy tends to offer slightly superior efficacy compared to single-agent therapy (13). The definitive survival benefit of chemotherapy remains unclear. Recent research into novel anti-angiogenic drugs suggests a potential alternative for improving upon the limited efficacy of conventional chemotherapy (14). In this case, upon recurrence with systemic metastasis, the initial chemotherapy regimen was doxorubicin plus docetaxel. Doxorubicin is a cornerstone first-line agent for advanced/metastatic soft tissue sarcoma. Given the patient’s multiple recurrences and rapid, widespread metastases indicating high aggressiveness, docetaxel was added as it is effective against various sarcomas and exerts strong inhibitory effects on highly proliferative tumors. After a single cycle, the therapeutic response was suboptimal, with enlargement of the sternal mass. Furthermore, both drugs carry a significant risk of myelosuppression, and the patient indeed developed grade IV myelosuppression, rendering the treatment intolerable. The regimen was subsequently changed to gemcitabine plus cisplatin. The mechanisms gemcitabine and cisplatin differ entirely from doxorubicin and docetaxel, making this an effective, non-cross-resistant second-line option following resistance to the first-line regimen. This combination also exhibits lower myelosuppressive toxicity. Anlotinib, a multi-target receptor tyrosine kinase inhibitor targeting VEGFR, PDGFR, FGFR, among others, exerts anti-angiogenic effect by inhibiting the formation of new tumor blood vessels. Anlotinib is officially approved in China for the second-line treatment of advanced soft tissue sarcoma. Consequently, the regimen was further adjusted to gemcitabine plus cisplatin plus anlotinib. The adjustments reflect the necessity for individualized selection and modification in the systemic therapy of MPT. Regrettably, after one cycle of the new regimen, the patient did not continue treatment. This prompts reflection: for PT with high-risk histological features or a clinically rapid progression/refractory course, genetic testing should be considered early to gain more information for diagnosis, prognosis, and identifying therapeutic targets, ultimately enabling more precise personalized treatment.

### 3.6 Molecular characteristics, prognostic markers, and exploration of precision therapy

#### 3.6.1 Genomic features and key prognostic markers

The MED12 gene is the most frequently mutated gene in fibroepithelial tumors, representing an early driver event in tumorigenesis (15, 16). However, the TERT promoter plays a more critical role in the progression to malignancy. The frequency of TERT promoter mutations increases significantly from benign to malignant PT, reaching up to 69.7% in MPT, and is considered a key driver of PT progression (16–18). Concurrently, alterations in classic oncogenes and tumor suppressor genes such as TP53, EGFR, and PIK3CA, directly participating in malignant transformation (17–19). These genomic alterations constitute the core molecular profile of PT and can be translated into practical prognostic markers through methods like immunohistochemistry. Ki-67 and p53: The Ki-67 proliferation index is an indicator of tumor aggressiveness. Abnormal p53 expression is a marker of lost TP53 gene function, associated with high-grade tumors and poor prognosis. The combined assessment of Ki-67 and p53 can effectively identify malignant types and result in adverse prognosis (15, 19). In this case, the Ki-67 index increased sharply from 20% after the initial surgery to 40% upon recurrence, accompanied by abnormal high expression of p53 (40%+). This combination not only far exceeded risk thresholds but also functionally revealed the essence of uncontrolled proliferation and genomic instability. MED12 and TERT: MED12 mutations are associated with better prognosis, while its wild-type status correlates with an increased risk of recurrence (19). TERT promoter mutation is a clear marker of progression and poor prognosis (18). The unknown genetic status in this case represents a significant information gap. Detection of MED12 wild-type coupled with TERT promoter mutation would perfectly explain the extreme aggressiveness at the genomic level. EGFR/PDGFR: EGFR amplification and mutation are among the malignant drivers in MPT and are associated with increased recurrence risk (18, 19). Stromal PDGFR β positivity is linked to increased disease-related mortality (19). These alterations are not only markers of poor prognosis but also potential drug targets.

#### 3.6.2 Indications for genomic testing and prospects for precision therapy

Based on the above molecular characteristics, genomic testing demonstrates crucial value for MPT. Key indications include: 1.Aiding Diagnosis: To assist in differentiating diagnostically challenging spindle cell tumors. 2. Prognostic Assessment and Risk Stratification: For tumors with high-risk clinical features, enabling precise risk stratification through the detection of genes. 3. Guiding Targeted and Immunotherapy for Advanced Patients. For patients with metastatic/refractory MPT, comprehensive genetic testing should be considered a standard procedure. This approach not only uncovers the molecular roots of its aggressiveness but also advances treatment from traditional, broad-spectrum chemotherapy into a new era of “precision strikes” based on specific molecular alterations, ultimately offering the potential for greater survival.

## 4 Conclusion

In summary, optimizing the management of MPT of the breast hinges on several key strategies: early and precise diagnosis, radical surgery aiming for wide negative margins, judicious use of adjuvant radiotherapy in high-risk patients, and exploration of individualized systemic therapy for advanced cases. Future efforts should focus on leveraging molecular technologies and conducting multi-center research to develop more effective and standardized diagnostic and therapeutic protocols.

## Data Availability

The original contributions presented in the study are included in the article/supplementary material. Further inquiries can be directed to the corresponding author.
